# IQ trajectories in autistic children through preadolescence

**DOI:** 10.1002/jcv2.12127

**Published:** 2023-01-31

**Authors:** Marjorie Solomon, An‐Chuen (Billy) Cho, Ana‐Maria Iosif, Brianna Heath, Apurv Srivastav, Christine Wu Nordahl, Emilio Ferrer, David Amaral

**Affiliations:** ^1^ Department of Psychiatry and Behavioral Sciences University of California‐Davis Sacramento California USA; ^2^ Imaging Research Center Sacramento California USA; ^3^ MIND Institute Sacramento California USA; ^4^ Department of Public Health Sciences University of California‐Davis Davis California USA; ^5^ Department of Psychology University of California‐Davis Davis California USA

**Keywords:** adolescence, autism, early childhood, intellectual development, IQ, outcomes, trajectories

## Abstract

**Background:**

We extended our study of trajectories of intellectual development of autistic individuals in early (mean age 3 years; T1), and middle childhood (mean age 5 years, 7 months; T2) into later middle childhood/preadolescence (mean age 11 years, 6 months; T3) in the longitudinal Autism Phenome Project cohort. Participants included 373 autistic children (115 females).

**Methods:**

Multivariate latent class growth analysis was used to identify distinct IQ trajectory subgroups. Baseline and developmental course group differences and predictors of trajectory membership were assessed using linear mixed effects models for repeated measures with pairwise testing, multinomial logistic regression models, and sensitivity analyses.

**Results:**

We isolated three IQ trajectory groups between T1 and T3 for autistic youth that were similar to those found in our prior work. These included a group with persistent intellectual disability (ID; 45%), a group with substantial increases in IQ (CHG; 39%), and a group with persistently average or above IQs (P‐High; 16%). By T3, the groups did not differ in ADOS‐2 calibrated severity scores (CSS), and there were no group differences between Vineland (VABS) communication scores in CHG and P‐High. T1‐T3 externalizing behaviors declined significantly for CHG, however, there were no significant T3 group differences between internalizing or externalizing symptoms. T1 correlates for CHG and P‐High versus ID group membership included higher VABS communication and lower ADOS‐2 CSS. A T1 to T2 increase in VABS communication scores and a decline in externalizing predicted CHG versus ID group membership, while T1 to T2 improvement in VABS communication and reduction in ADOS‐2 CSS predicted P‐High versus ID group membership.

**Conclusions:**

Autistic youth exhibit consistent IQ developmental trajectories from early childhood through preadolescence. Factors associated with trajectory group membership may provide clues about prognosis, and the need for treatments that improve adaptive communication and externalizing symptoms.

## Graphical abstract

Three IQ trajectories between early childhood and preadolescence were isolated for autistic youth. One showed persistent intellectual disability (ID), one showed persistent higher intellectual ability, and one demonstrated increasing intellectual development with IQ scores rising at least 1 standard deviations. Repetitive behaviors and externalizing behaviors declined for this group with increasing IQs, who together with the persistently high group showed greater symptoms of internalizing. The group with persistent ID showed the most impaired communication and social adaptive functioning, and an NVIQ that was significantly greater than VIQ in early childhood.

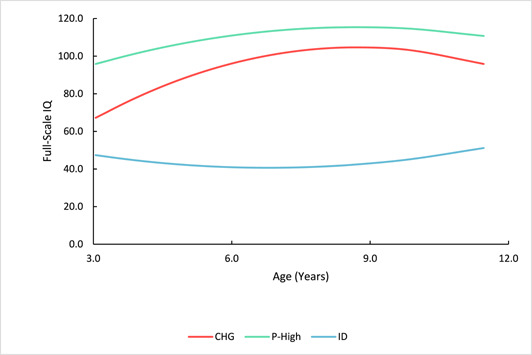

**Key points**

- In this study of the intellectual development of autistic individuals from early childhood through age 12, we found there were three IQ trajectories—a group with intellectual disability from early childhood through preadolescence (ID; 45%), a group whose IQs increased at least 1 standard deviation referred to as Changers (CHG; 39%) and a group whose IQs were in the average or above range through the period (P‐High; 16%).
- Although autistic youth exhibited lower adaptive functioning than would be expected based on IQ. By preadolescence, there were no significant group differences between adaptive communication in the CHG or P‐High groups.
- Early correlates for being in the CHG or P‐High groups versus the ID group, included stronger early VABS communication scores and lower ADOS CSS.
- Improved communication adaptive functioning and decreased externalizing between T1 and T2 was a marker of becoming a member of CHG versus ID, while reduced ADOS‐2 CSS and improved adaptive communication were predictive of being in P‐High versus ID.
- Findings suggest that early communication adaptive functioning and may be a stronger prognostic marker than IQ scores, and that communication adaptive functioning and externalizing symptoms may be treatment targets that are associated with later improvements in intellectual ability levels.

## INTRODUCTION

Given the heterogeneity of autism (Geschwind & Levitt, 2007), it remains difficult to provide reliable answers about what the future holds for young autistic children. Some never acquire functional spoken language, sustain close interpersonal relationships outside of family members or caregivers, or live independently. Others develop meaningful reciprocal friendships, obtain post‐secondary education, and work and live in the community (Mason et al., 2021). Some even “lose” their autism diagnoses (Fein et al., 2013). Intellectual ability level, as assessed using IQ or a developmental quotient (DQ) (both referred to as IQ) is perhaps the most significant predictor of outcomes across key life domains for autistic individuals (Miller & Ozonoff, 2000; Munson et al., 2008). Early IQ also is the strongest predictor of adult outcomes in autistic individuals (Pickles et al., 2020).

IQ typically increases through early childhood in autistic children (Eaves & Ho, 2008; Flanagan et al., 2015), with the most rapid growth during the first 6–7 years of life (Pickles et al., 2014). Nonverbal IQ (NVIQ) and verbal IQ (VIQ) show comparable development (Anderson et al., 2014; Solomon et al., 2018). IQ has been associated with adaptive functioning that is typically lower than IQ (Franchini et al., 2018), social cognition (Hirosawa et al., 2020), autism symptom severity (Pickles et al., 2020), and psychopathology (Charman et al., 2017). Finally, those losing their diagnosis have higher childhood IQs (Fein et al., 2013).

While there have been multiple studies examining the association between intellectual functioning in childhood and later outcomes, few have been longitudinal and fewer still have investigated IQ‐based subgroups/phenotypes using data‐driven or clinically‐based clustering strategies. A first study to isolate IQ‐based subgroups using data‐driven methods idenified four unique groups based on IQ level and relative strength of verbal versus non‐verbal abilities in 2–5 1/2 year olds (Munson et al., 2008). Two subsequent studies employed clinical grouping methods. The first examined a prospective longitudinal cohort of 85 children assessed at 2, 3, and 19 years (*n* = 85). They used age‐19 IQ to group participants into VIQ<70 and VIQ>70 sub‐groups who did and did not retain their diagnoses. Eighty five percent of the group remaining intellectually disabled could be identified from early IQ scores. Participants losing their autism diagnosis received more early intervention and exhibited early reduction in restricted and repetitive behaviors (Anderson et al., 2014). The second study using a clinical grouping approach assessed participants at ages 2 and 13 years, assigned children to a best outcomes (IQ > 80 with no diagnosis of autism by the second assessment; 16%), more able (IQ > 80 throughout, 20%), and more challenged (IQ<80%; 63%) groups (Zachor & Ben‐Itzchak, 2020). The more challenged group showed decreased cognitive ability and increased social and repetitive behavior severity over time.

To the best of our knowledge, a study by our group has been the only prospective longitudinal study to use an empirical data‐driven approach to isolate developmental trajectories of intellectual functioning in children as young as ages 2–8 years old (Solomon et al., 2018). Four distinct groups were identified. Two had persistent intellectual disability (ID) (43% of the sample), 1 had IQs starting in the intellectual disability range that then increased by at least 2 standard deviations (35%), and 1 had IQs remaining in the average or better range over time (22%). Communication and social adaptive functioning lagged IQ in all autism but not non‐autistic groups. While internalizing symptoms decreased over time for all groups, externalizing symptoms declined only for the group experiencing substantial increases in IQ.

The current study aims to extend our past investigation of trajectories of IQ development in one of the few relatively large, cognitively heterogeneous, and recent longitudinal cohorts—the Autism Phenome Project (APP)—by adding a third data point from our middle childhood assessment and by investigating additional developmental issues pertinent to the preadolescent developmental period. We again isolate phenotype groups based on IQ and characterize them based on autism symptoms, communication adaptive functioning, and problem behavior symptoms including internalizing and externalizing. To gain insight on predictors of later childhood/pre‐adolescent outcomes, we then investigate variables assessed at or before T1 and changes between variables assessed at T1 and T2. These analyses focus on group differences in potential predictors for children who remained in the ID group versus those who did not by T3.

## METHOD

### Participants

Participants were members of the longitudinal APP cohort, which began recruiting both autistic and typically developing children through an internal data base and advertisements placed with local providers and other organizations and groups known to be involved with young autistic children and their families starting in 2006. Baseline assessments were conducted in children at 2–5 years of age, followed by longitudinal assessments across childhood. Four total assessments have been completed. A fifth is in progress and the cohort has been expanded. To increase female representation within the APP cohort, we initiated the Girls with Autism—Imaging of Neurodevelopment (GAIN) study in 2014. All participants in the GAIN study are automatically included in the APP dataset. This explains why the gender ratio in new participants is enriched for females. Inclusion criteria for autism were based on the NIH Collaborative Programs of Excellence in Autism as described in our prior study (Solomon et al., 2018). Although the full cohort included TD children, we examined IQ trajectory classes *within* the autistic group and thus excluded TD participants from analyses.

IQ/DQ assessments were completed at three of these assessment points, which we refer to as T1 (mean age = 3.0 years, SD = 0.5, *n* = 373); T2 (mean age = 5.6 years, SD = 0.9, *n* = 154); and T3 (mean age = 11.5 years, SD = 0.9, *n* = 116). One hundred and eighty‐two autistic participants had IQ data only at T1, 112 participants had data at two timepoints (T1 and T2: 75, T1 and T3: 37), and 79 participants had data at all three timepoints. See Table 1 for a summary of demographic and clinical characteristics including the IQ scores of the entire sample across the three assessments. We included all autistic APP participants with IQ data at T1 in our analyses. Supplementary Table S1 compares the demographic and clinical characteristics of the participants with complete data versus those with only 1 follow‐up visit and those with only baseline data to illustrate their similarity to the entire sample. We did not find a systematic pattern of IQ differences for children having fewer visits as compared to those with complete data. The only other observed characteristic significantly related to missingness was sex, because the most recent participants were from the GAIN cohort. Thus, sex was included as a covariate in all models.

**TABLE 1:** Demographics and clinical characteristics of the participants at T1, T2, and T3

Characteristic	T1	T2	T3
	(*n* = 373)	(*n* = 154)	(*n* = 116)
Male sex, *n* (%)	258 (69.2%)	102 (66%)	92 (79.3%)
ADOS module completed,^a^ *n* (%)			
Module 1	315 (84.5%)	51 (33.1%)	31 (27.0%)
Module 2	58 (15.5%)	42 (27.3%)	12 (10.4%)
Module 3	–	61 (39.6%)	72 (62.6%)
ADOS‐2 calibrated severity score,^a^ mean (SD)	7.5 (1.7)	7.0 (2.1)	7.6 (1.9)
Age (years) at IQ testing, mean (SD) [range]	3.0 (0.5)	5.6 (0.9)	11.5 (0.9)
	[1.7–5.0]	[4.3–9.4]	[9.0–13.6]
IQ^b^			
Full scale,^c^ ^,^ ^d^ mean (SD) [range]	62.7 (20.7)	79.1 (32.3)	78.8 (32.1)
	[20.5–132.5]	[17.9–133.0]	[24 – 170.0]
Verbal,^d^ mean (SD) [range]	55.6 (25.0)	74.8 (33.0)	73.2 (36.0)
	[14.0–128.0]	[12.1–128.0]	[24 – 157.0]
Nonverbal,^e^ mean (SD) [range]	69.9 (18.7)	82.2 (31.2)	82.4 (30.4)
	[25.5–137.0]	[22.3–140.0]	[24 – 170.0]
VABS communication,^f^ mean (SD)	72.9 (15.7)	81.0 (19.7)	72.8 (18.3)
CBCL internalizing,^g^ mean (SD)	62.2 (9.5)	59.4 (9.7)	60.0 (10.1)
CBCL externalizing,^h^ mean (SD)	59.3 (10.9)	55.8 (10.3)	55.1 (9.5)

Abbreviations: ADOS‐2, Autism Diagnostic Observation Schedule; CBCL, Child Behavior Checklist; Second, Edition; VABS, Vineland Adaptive Behavioral Scales.

^a^ 1 participant ADOS was not completed at T3.

^b^ All participants had IQ assessed using MSEL at T1; at T2, 28 participants were assessed using MSEL and 126 using Differential Abilities Scales‐II (DAS‐II) (DAS); at T3, all participants were assessed using DAS.

^c^ 1 participant's IQ could not be assessed at T2.

^d^ 1 participant's IQ could not be assessed due to aggressive behavior at T3.

^e^ 2 participants' IQs could not be computed because nonverbal scores were out of range of the publisher's norms.

^f^ 31 participants at T1, 21 at T2, and 6 at T3 are missing this variable.

^g^ 49 participants at T1, 34 at T2, and 13 at T3 are missing this variable.

^h^ 43 participants at T1, 26 at T2, and 12 at T3 are missing this variable.

**TABLE 2:** Summary of the multinomial logistic regression models predicting subgroup membership using early correlates, intervention intensity, and T1 to T2 behavioral changes

	CHG versus ID			P‐High versus ID		
	Estimate	OR [95% CI]	*p*‐value	Estimate	OR [95% CI]	*p*‐value
Early correlate (T1)						
ADOS‐2 CSS	−0.33	0.72 [0.61–0.83]	<0.001	−0.51	0.60 [0.50–0.73]	<0.001
VABS communication	0.11	1.12 [1.09–1.15]	<0.001	0.19	1.21 [1.16–1.26]	<0.001
CBCL internalizing	−0.003	1.00 [0.97–1.02]	0.83	0.006	1.01 [0.97–1.04]	0.75
CBCL externalizing	0.02	1.02 [0.999–1.04]	0.06	−0.0001	1.00 [0.97–1.03]	0.99
Total intensity of services received	−0.05	0.95 [0.75–1.20]	0.66	−0.04	0.96 [0.69–1.35]	0.83
T1 to T2 behavioral change						
ADOS‐2 CSS	−0.08	0.93 [0.77–1.12]	0.43	−0.31	0.73 [0.58–0.92]	0.007
VABS communication	0.08	1.08 [1.04–1.12]	<0.001	0.05	1.05 [1.01–1.09]	0.02
CBCL internalizing	−0.05	0.95 [0.91–1.00]	0.052	−0.01	0.99 [0.94–1.04]	0.57
CBCL externalizing	−0.08	0.93 [0.88–0.97]	0.001	−0.02	0.98 [0.94–1.03]	0.37

*Note*: Early correlates are defined as scores at T1; T1 to T2 Behavior Change is defined as the difference between the score at T2 and the score at T1. Models were adjusted for child sex and age. Estimates and ORs are reported for a one‐unit increase in the predictor for all variables except intensity of services received; for this variable, estimates and OR are reported for a one‐standard‐deviation increase.

Abbreviations: ADOS‐2, CSS; Autism, Diagnostic Observation Schedule Calibrated Severity Score; CBCL, Child Behavior Checklist; CHG, Changers; CI, Confidence Interval; ID, Persistent Intellectual Disability; OR, Odds Ratio; P‐High, Persistently High IQ; VABS, Vineland Adaptive Behavioral Scales.

The study has been approved by the University of California Davis Institutional Review Board, with parents or legal guardians providing informed consent. Data is available upon request from the corresponding author.

### Measures

IQ‐measures used include the MSEL and the Differential Abilities Scales‐II (DAS‐II; (Elliot, 2007)). To characterize the IQ‐based subgroups, the ADOS and the ADOS‐2, when it became available, (Gotham et al., 2007), which was administered at each time point, the ADI‐R (Lord et al., 1994), Vineland Adaptive Behavior Scales, Second Edition: (Parent/Caregiver Rating Form) (VABS‐2) (Sparrow et al., 2005), Child Behavior Checklist (CBCL)‐Preschool (Achenbach & Rescorla, 2000) and School‐Age (Achenbach & Rescorla, 2001) versions were used. All diagnostic and cognitive assessments were conducted by licensed clinical psychologists specializing in autism and trained according to research standards for these tools. At each visit, caregivers also completed a record adapted from the Collaborative Programs of Excellence in Autism that inquired about the type and duration of current and previous interventions received. See the Supporting Information for measurement descriptions.

### Statistical analyses

We first identified distinct IQ‐based subgroups and their differential developmental trajectories by conducting a latent class growth analysis (LCGA) of autistic participants' full‐scale IQ scores using Mplus 8 (Muthen, 2017). All participants with at least one timepoint were included (*n* = 373), and both linear and quadratic age‐based models were evaluated for best fit. Models were estimated using full‐information maximum likelihood, which permitted us to include the participants with missing data, under the missing‐at‐random assumption. Information‐heuristic (e.g., information criterion values) and inferential (e.g., likelihood ratio tests) relative fit comparisons were used to select the best‐fitting solution. Information‐heuristic indices include the Akaike Information Criterion (AIC), Bayesian Information Criterion (BIC), and sample size‐adjusted BIC (SBIC), for which lower values indicate better fit, as well as the approximate Bayes Factor (BF) (Wasserman, 2000). BF compares a larger model with a smaller one and a higher score indicates the larger model is the more probable correct model (values between 1‐3 represent weak, 3–10 moderate, and >10 strong evidence for the larger model). As an inferential index, we used the approximate correct model probability (CMP) (Schwarz, 1978), which compares a single model versus all other models under consideration; models with a CMP >0.10 should be considered as candidate models. We used the highest posterior probability from the best fitting model to assign each participant to their most likely subgroup.

Next, we examined differences in trajectories of clinical characteristics for the identified subgroups using linear mixed effects models (Laird & Ware, 1982) implemented in SAS 9.4 (SAS Institute Inc., 2002–2012). For each clinical characteristic, we began with a model that included fixed effects for latent class group, linear and quadratic effects of age (centered at 3 years), sex, as well as age by latent class group interactions. Higher‐level interactions were tested and not retained in the reported model if they did not contribute significantly. The models also included random effects for intercept and linear and quadratic effects of age, to account for the within‐child dependence. Following significant overall tests for the interaction between latent class group interactions and age, we examined pairwise differences between latent class groups at the average age at each visit, controlling for multiple comparisons (Tukey‐Kramer adjustment). Similarly, linear contrasts were constructed to examine whether changes from average age of the sample at T1 (3.0 years) to average age of the sample at T3 (11.5 years) were significant within each subgroup.

Multinomial logistic regression was used to examine T1 correlates and behavioral variable changes between T1 and T2 and their associations with trajectory membership, after adjusting for sex and age at testing.

Finally, because using maximal probability ignores the uncertainty in class assignment, we conducted a sensitivity analysis by using multiple pseudo‐class draws (Bandeen‐Roche et al., 1997), when examining differences in trajectories of clinical characteristics across latent classes. Children were randomly classified into latent classes 100 times based on their distribution of posterior probabilities from the best fitting LCGA model. The subsequent analyses were performed 100 times (i.e., for each draw) and results were combined across draws using standard methods for multiple imputation for missing data (Rubin, 1987). The same strategy was employed to examine the robustness of the predictors of trajectory membership.

## RESULTS

At T1, a significant proportion of autistic participants achieved the lowest possible MSEL standard score so verbal, nonverbal, and full‐scale (i.e., combined) IQ were estimated by calculating ratio developmental quotient scores by dividing average verbal, nonverbal, and combined MSEL subscale age equivalents by chronological age and then multiplying by 100. At T2 and T3, DAS‐II verbal cluster standard score, special nonverbal composite and general conceptual ability scores were used within analyses as verbal (VIQ), non‐verbal (NVIQ), and full‐scale IQ FSIQ estimates. At T2, children who were unable to achieve basal scores on the DAS‐II (*n* = 28) were administered the MSEL, and developmental quotients were used to provide nonverbal, verbal, and combined IQ estimates. At T3, if children were unable to complete the School Age form, the Early Years form was used (*n* = 24).

(1) Full‐scale IQ Trajectories: Both linear and quadratic models were considered within the LCGA for the autistic participants. Fit indices for one‐class to four‐class solutions for the quadratic models are summarized in Table S2. A quadratic model with three classes emerged as the best‐fitting model, being supported by multiple indices, including BIC and SBIC. AIC was the only index supporting a four‐class solution, although the values for the three‐ (5601.6) and four‐class solutions (5599) were similar. Moreover, the four‐class model identified a class that included <5% of the sample. Thus, the three‐class solution was selected as the best fit.

**FIGURE 1:**
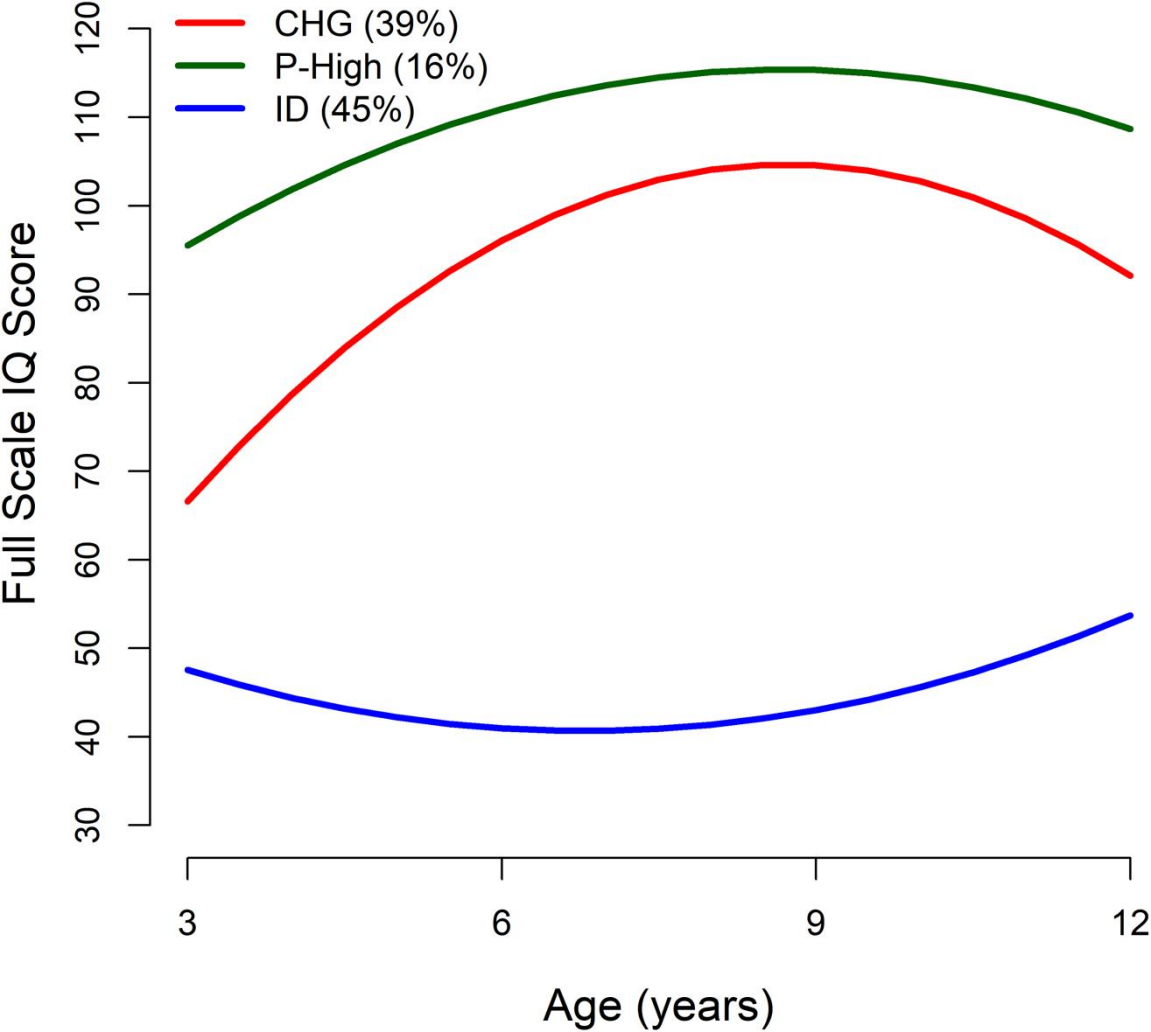
IQ trajectories of the three full‐scale IQ subgroups: Changers (CHG), persistently high IQ (P‐High) and persistent intellectual disability (ID).

Using the best‐fitting model, participants were then classified into the subgroups where they presented the highest posterior classification probability. One subgroup (*n* = 147 [39%]; labeled “Changers” [CHG]) began with notably low IQs followed by a substantial increase that slowed as they entered middle childhood/preadolescence. The trajectory for the second subgroup (*n* = 167 [45%]; “Persistent Intellectual Disability” [ID]) also began with a low IQ that persisted across childhood. The last subgroup (*n* = 59 [16%]; “Persistently High IQ” [P‐High]) presented a trajectory that demonstrated relative stability with a gradual increase during childhood. See Figure 1. The average assignment probabilities for the subgroup classes were 0.80, 0.85, and 0.86, respectively. Group membership was very similar to that identified in our previous manuscript using data from T1 and T2 only (Table S3). Demographic and clinical characteristics for all subgroups across the three timepoints are presented in Table S4.

**FIGURE 2:**
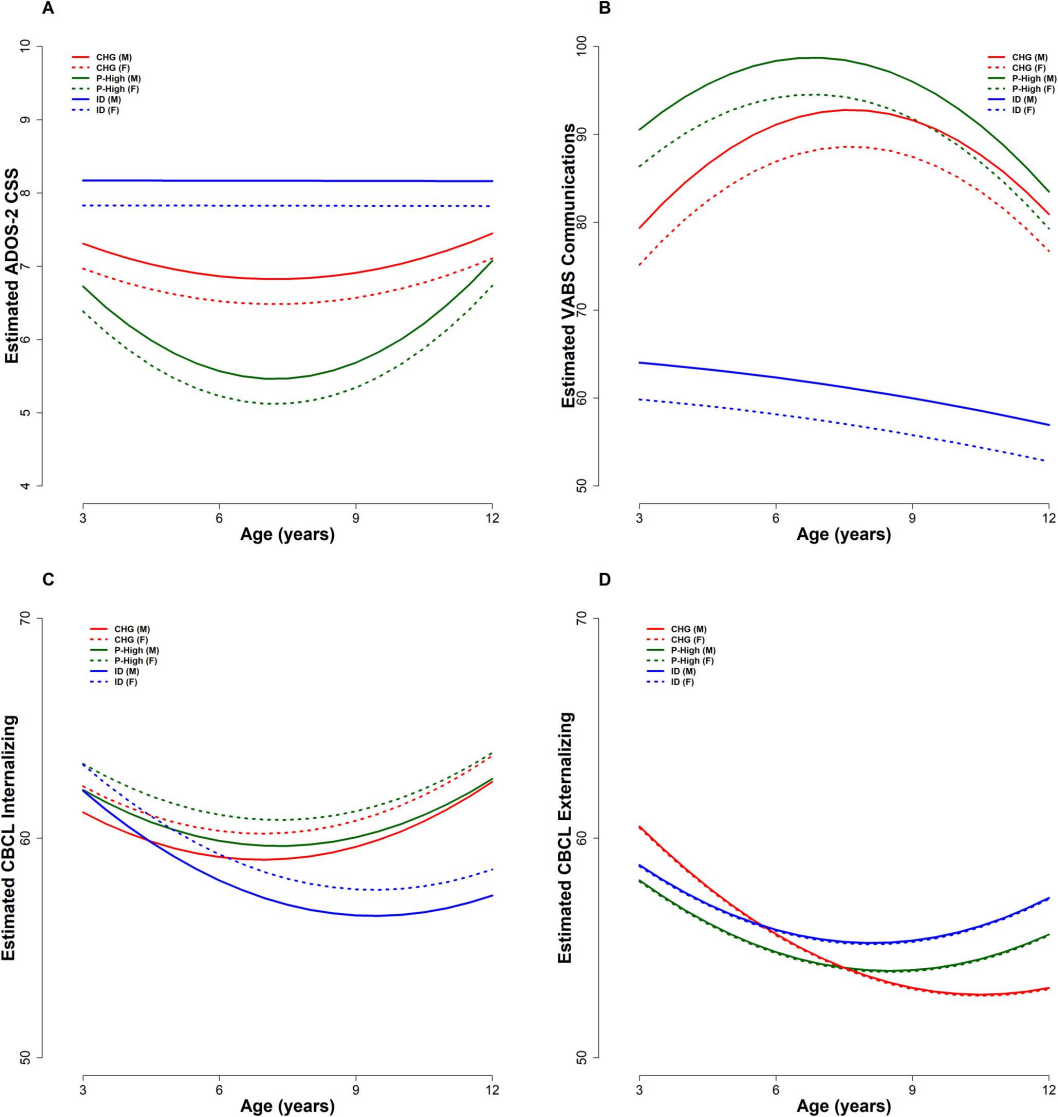
Trajectories of ADOS‐2 Calibrated Severity Score (A), Vineland Communication (B), CBCL Internalizing T‐Scores (C) and Externalizing T‐Scores (D) by IQ trajectory subgroup and sex. CHG, Changers; F, Female; ID, Persistent Intellectual Disability; M, Male; P‐High, Persistently High IQ.

To affirm that the IQ increases of the CHG and other groups did not simply reflect language acquisition and the consequent increase in VIQ, we examined those participants with FSIQ changes of 15 points or more (1 standard deviation) from T1 to T3. Notably, for CHG, 89% also showed increases in NVIQ, while 84% experienced changes in both VIQ and NVIQ (Table S5). We also completed trajectory analyses using NVIQ and VIQ. Here we found that 87.8% of those categorized in CHG in the current analysis would continue to be if NVIQ were used. These percentages were 55.9% for P‐High and 82.6% for ID. Values were all over 80% when VIQ was used (Table S6).

(2) ADOS‐2 Calibrated Severity Score (CSS): Parameter estimates for all mixed‐effects models fitted to clinical variables and adjusted for sex are summarized in Table S7. In the CHG and P‐High groups, ADOS‐2 CSS decreased from T1 to T2 although it returned to the T1 levels by late middle childhood/preadolescence (T1 vs. T3, CHG: *p* = 0.95; P‐High: *p* = 0.94). For the ID group, ADOS‐2 CSS scores remained consistent from T1 to T3 (*p* = 0.98). By T3, the three groups did not differ in ADOS‐2 CSS. See Figure 2A.

The developmental pattern of autism symptom severity change has been studied previously by our group in a smaller sample not including all data points (Waizbard‐Bartov et al., 2022). The current results did not overlap with this other study given that there were no significant associations between IQ trajectory membership and their three groups (defined by increasing, decreasing, and stable calibrated ADOS‐2 CSS). See Table S8.

(3) Communication Adaptive Functioning: From T1 to T3, CHG significantly increased in VABS communication score (*p* = 0.03), while ID decreased and P‐High remained relatively stable (ID: *p* < 0.001, P‐High: *p* = 0.11; Figure 2B). Thus, while differences between the three subgroups were present at T1, CHG and P‐High showed no communication score differences by T3 (*p* = 0.66), despite their being significantly higher than ID (both *p* < 0.001).
(4) Internalizing and Externalizing Symptoms: The three autistic subgroups had similar CBCL internalizing subscale scores at T1. By T3, the score for the ID group decreased, although this reduction was not significantly different than that found in the other groups, and there were no group differences in scores at T3 (after adjusting for multiple comparisons, all *p* > 0.06, Figure 2C). On the externalizing subscale, the three groups also had comparable scores at T1. The CHG group showed a significant externalizing score decline from T1 to T3 (*p* < 0.001), however, here too, none of the groups differed on this variable at T3 (after adjusting for multiple comparisons, all *p* > 0.14, Figure 2D).
(5) Demographic Characteristics, and Loss of Diagnosis: The three autism subgroups did not differ in sex composition or maternal and paternal age at childbirth (Table S3). The groups did not differ in the total hours of services they received or the intensity of these services. Of the 191 participants (CHG: 72; P‐High: 38; ID: 81) with at least two timepoints, 10 lost their autism diagnosis as defined by the ADOS‐2 CSS. Of those with at least two timepoints, 8.3% of the CHG group and 10.5% of the P‐High group lost their autism diagnoses as assessed by this same criteria, compared to none of the participants in the ID group (*p* < 0.001; Table S3).
(6) Multinomial Regression Analyses: Multinomial logistic regression was used to assess the predictive value of early correlates (at or before T1) and behavioral variables changing from T1 to T2 on IQ subgroup membership at T3 (Table 2). Models examined both CHG and P‐High compared with ID. Higher T1 scores on the VABS communication scale (*p* < 0.001) and lower scores on the ADOS‐2 CSS, as well as T1 to T2 increases in the VABS communication scale score (*p* < 0.001) and decreases in the CBCL externalizing score (*p* < 0.002), predicted being in the CHG versus the ID group. More favorable T1 scores on the ADOS‐2 CSS (*p* < 0.001) and the VABS communication scale (*p* < 0.001), as well as T1 to T2 improvements in the VABS communication scale (*p* = 0.02) and ADOS‐2 CSS (*p* = 0.007) predicted being in P‐High versus ID.

Sensitivity analysis results (Supplementary Tables S9 and S10) supported the primary analyses. While the magnitude of the estimates generally slightly decreased after accounting for uncertainty in group assignment, all primary analysis findings remained significant.

## DISCUSSION

We extended the study of the trajectories of intellectual development of autistic individuals into late middle childhood/preadolescence in the cognitively heterogeneous APP cohort. Consistent with our prior work, autistic participants were assigned a group with intellectual disability from early childhood through preadolescence (ID; 45%), a group whose IQs increased substantially during early childhood referred to as Changers (CHG; 39%) or a group whose IQs were in the average or above range through the period (P‐High; 16%). Unlike our prior study where P‐High ADOS‐2 CSS scores declined, the new groups did not differ with respect to autism severity at T3. Between middle childhood and preadolescence, VABS communication scores increased in CHG, decreased in ID, and stayed the same in P‐High, such that there were no T3 group differences between CHG and P‐High. T1‐T3 externalizing declined significantly for CHG, although, there were no T3 group differences for internalizing or externalizing. T1 correlates for CHG and P‐High versus ID group membership at T3 included higher VABS communication and lower ADOS‐2 CSS. A T1 to T2 increase in VABS communication scores and a decline in externalizing predicted CHG versus ID group membership at T3, while a T1 to T2 improvement in VABS communication and a reduction in ADOS‐2 CSS predicted P‐High versus ID group membership.

The rapid IQ gains in the CHG group we found in prior work slowed after middle childhood. While this is not consistent with two recent studies that report average mean IQ improvements through adolescence (Prigge et al., 2021; Simonoff et al., 2020), these studies examined mean differences versus trajectories, and Prigge et al. investigated only intellectually able participants. Also noteworthy is that the positive T1‐T2 autism symptom severity and communication adaptive functioning changes in CHG and P‐High also slowed between T2 and T3. Waizbard and colleagues observed a similar pattern when they focused on autism symptom severity (Waizbard‐Bartov et al., 2022). While we cannot entirely rule out that the reversion back to original scores was a statistical artifact, this pattern was not present for all measures or groups, providing support for a true reversion. Perhaps the complexity of social and cognitive developmental tasks of early adolescence expose more autism related traits, resulting in relative skill declines. In fact there is a growing consensus that the period of transition to school may be a turning point in autistic development (Georgiades et al., 2022) with age 6 representing a time of plateauing in early symptom improvement.

Only the CHG group experienced significant reductions in externalizing symptoms between T1 and T3. While internalizing scores in P‐High and CHG did not increase with the beginning of adolescence as might be expected (Solomon et al., 2012), the ID group experienced some reduction in these symptoms, as has been found by others (Edirisooriya et al., 2021). However, it is not clear that internalizing symptoms, and especially anxiety, can be well measured for children with intellectual disability (Kerns et al., 2021), so these findings must be interpreted with caution.

This work provides several clinically significant findings. Related to prognosis, we found that both lower T1 ADOS‐2 CSS scores and higher VABS communication scores were associated with being in either the CHG or P‐High versus ID group by T3. Here, it is especially striking that the VABS—a common inexpensive parent reported measurement—offered a better early indicator of what the future held than an early clinician administered IQ test. We also would point out that, while IQ and adaptive functioning are typically correlated, there is a sizable body of literature suggesting that IQ and adaptive functioning are not necessarily so tightly associated in autistic samples (Duncan & Bishop, 2015). In fact, in our sample, although T1 IQ and the VABS communication were highly correlated overall (*r* = 0.70), correlations between the VABS and IQ differed substantially for the trajectory groups, ranging from *r* = 0.5 for ID; 0.46 for P‐High; to 0.25 for CHG.

Another clinically interesting observation with prognostic implications was that, contrary to popular clinical belief, language acquisition and VIQ change were not the sole drivers of overall intellectual development. Instead, we found that for CHG, 89% showed increases in NVIQ, while 84% experienced changes in both VIQ and NVIQ (Table S4). Thus, NVIQ did not become stable by age 3 in most participants, and even individuals with moderate mental disability could become members of CHG.

A second set of clinically and potentially intervention‐relevant markers were those associated with T1‐T2 changes. Here we found that T1‐T3 increases in VABS communication scores rendered the CHG and P‐High groups equivalent by T3 and distinguished both from ID. We also found that T1‐T2 increases in externalizing symptoms were more characteristic of the CHG versus the ID group, and that T1‐T2 decreases in ADOS CSS were more characteristic of participants in the P‐High group. Although the precise cause and effect associations between IQ, adaptive functioning, externalizing, and autism symptoms remain unclear, our results suggest that each of these areas can improve. Furthermore, they may be critical treatment targets that drive the development of intellectual functioning, and fortunately, effective interventions in these areas have been developed (Kenworthy et al., 2014; Kim et al., 2021; Solomon et al., 2008).

This study had several limitations. First, some have shown that the DAS and MSEL are not entirely comparable, especially in the middle IQ ranges (Farmer et al., 2016). Although, others find no systematic differences (Bishop et al., 2011). Additionally, neither measure does a good job of assessing profound intellectual disability, requiring us to use floor scores for 28 participants. Second, while the LCGA and linear mixed‐effects models used were able to handle missing data and produce valid results in the presence of data missing at random, their results may be biased if missingness depends on the missing values themselves. While formally testing whether the assumption of missingness at random (MAR) holds would require data from non‐responders, our examination of missingness thus far suggests that MAR may be a plausible assumption here. Finally, by focusing on IQ, we adopted a very narrow definition of future outcomes. Recent studies have rightly encouraged broadening the meaning of outcomes (Lord et al., 2020; Mason et al., 2021).

In conclusion, we showed that autistic youth from our middle childhood assessment continued to display IQ trajectories that were similar to those we observed earlier in childhood. We identified early and ongoing correlates of late middle childhood/preadolescence outcome which hold the potential to provide critical information related to prognosis and treatment development.

## AUTHOR CONTRIBUTIONS

**Marjorie Solomon**: Conceptualization; Data curation; Funding acquisition; Investigation; Methodology; Project administration; Resources; Supervision; Writing – original draft; Writing – review & editing. **An‐Chuen (Billy) Cho**: Conceptualization; Data curation; Formal analysis; Methodology; Writing – original draft; Writing – review & editing. **Ana‐Maria Iosif**: Conceptualization; Data curation; Formal analysis; Methodology; Supervision; Writing – original draft; Writing – review & editing. **Brianna Heath**: Data curation; Investigation; Project administration; Writing – original draft; Writing – review & editing. **Apurv Srivastav**: Formal analysis; Writing – review & editing. **Christine Wu Nordahl**: Conceptualization; Data curation; Investigation; Writing – original draft; Writing – review & editing. **Emilio Ferrer**: Conceptualization; Formal analysis; Investigation; Software; Supervision; Writing – original draft; Writing – review & editing. **David Amaral**: Conceptualization; Funding acquisition; Investigation; Writing – original draft; Writing – review & editing.

## CONFLICTS OF INTEREST

Drs. Solomon, Iosif, Nordahl, Cho, Heath, and Ferrer and Mr. Srivastav report no biomedical financial interests or potential conflicts of interest. Dr. Amaral is on the Scientific Advisory Board of Stemina Biomaker Discovery and Axial Biotherapeutics.

## ethical considerations

The study has been approved by the University of California Davis Institutional Review Board, with parents or legal guardians providing informed consent.

## Supporting information

Supporting Information S1

## Data Availability

Data openly available in a public repository that issues datasets with DOIs.
