# Supplementary material for: IQ trajectories in autistic children through preadolescence
Source: JCPP Adv. 2023 Jan 31;3(1):e12127. doi: 10.1002/jcv2.12127 (PMC10241474; doi:10.1002/jcv2.12127)
Supplement: Supplementary file 1 — Supporting Information S1 [file JCV2-3-e12127-s001.docx]

**Supporting Information**

IQ trajectories in autistic children through preadolescence

Marjorie Solomon ^1,2,3^, An-Chuen (Billy) Cho^1,3^, Ana-Maria Iosif^4^, Brianna Heath^1,3^, Apurv Srivastav^4^, Christine Wu Nordahl^1,3^, Emilio Ferrer^5^, David Amaral^1,3^

^1^Department of Psychiatry & Behavioral Sciences, University of California-Davis, Sacramento,

CA, 2230 Stockton Blvd., Sacramento, CA 95817

^2^Imaging Research Center, 4701 X Street, Sacramento, CA 95817

^3^MIND Institute, 2825 50^th^ Street Sacramento, CA 95817

^4^Department of Public Health Sciences, University of California-Davis, Davis, CA, One Shields

Ave., Davis, CA 95616

^5^Department of Psychology, University of California-Davis, Davis, CA, One Shields

Ave., Davis, CA 95616

Measure Descriptions

Descriptions of measures used in the study follows.

Mullen Scales of Early Learning (MSEL) ([Mullen, 1995](#_ENREF_25)). Cognitive and developmental functioning was measured using the MSEL at study entry. The MSEL is a standardized measure of cognitive and developmental functioning for children 0-68 months of age. MSEL yields subscale standard scores, age-equivalents, and composite standard scores. Four subscales were administered to provide measures of nonverbal (Visual Reception, Fine Motor) and verbal abilities (Expressive and Receptive Language). Since a significant proportion of the autism group achieved the lowest possible MSEL standard score, ratio developmental quotients (mental age/chronological age *100) were calculated to provide nonverbal, verbal and combined IQ estimates.

The Differential Abilities Scales-II (DAS-II) ([Elliot, 2007](#_ENREF_8)). The DAS-II is a standardized measure of cognitive abilities for children ages 2.5-17 years. Starting a T2, participants completed the core battery of the DAS-II Early Years or School Age form, which consists of verbal, nonverbal, and spatial reasoning clusters. This produced standardized cluster scores, a cognitive composite (General Conceptual Ability, GCA) and a combined nonverbal and spatial ability composite (Special Nonverbal Composite; SNC). DAS-II verbal cluster, SNC and GCA scores provided estimates of verbal, nonverbal and combined IQ. Children who were unable to achieve basal scores on the DAS-II (*n*=18) were administered the MSEL at T2, and development quotients were used to provide nonverbal, verbal, and combined IQ estimates. At T3, if children were unable to complete the School Age form, the Early Years form was used.

The Autism Diagnostic Observation Schedule (ADOS) and ADOS-2. Both are semi-structured standardized observations. Diagnostic classification is based upon exceeding a threshold in a combined Social Affect and Restricted and Restricted and Repetitive Behavior score. Calibrated severity scores (CSS) provide a common metric for comparison of scores across ADOS modules, yielding estimates of overall autism symptom severity ([Gotham, Pickles, & Lord, 2009](#_ENREF_14)) as well as separate social affect and restricted and repetitive behavior scores ([Hus, Gotham, & Lord, 2014](#_ENREF_17)). CSS range from 1-10 (1-3 Non-Autism; 4-5 Autism Spectrum; 6-10 Autism).

Autism Diagnostic Interview-Revised (ADI-R; ([C. Lord et al., 1994](#_ENREF_22))): This comprehensive parent interview probes for symptoms of AUTISM. It is administered by a trained clinician using a semi-structured interview format. ADI-R elicits information on over 100 questions about the child’s current behavior and developmental history. The significant developmental time point in the ADI-R is age 4 to 5 years; research indicates behaviors are at their peak by this age, making it a most sensitive time for identification. The items that empirically distinguish individuals with autism from those with other developmental delays are summed into three algorithm scores -- social difficulties, communication deficits, and repetitive behaviors.

Vineland Adaptive Behavior Scales, Second Edition: Parent/Caregiver Rating Form) (VABS-2) ([Sparrow et al., 2005](#_ENREF_33)). The Parent/Caregiver Rating Form was completed by caregivers to assess adaptive behavior in Communication, Daily Living Skills and Socialization domains. The Communication and social domain scores were used in our analysis.

Child Behavior Checklist (CBCL)-Preschool ([Achenbach & Rescorla, 2000](#_ENREF_2)) and School-Age ([Achenbach & Rescorla, 2001](#_ENREF_1)) versions. The CBCL, which is a part of the Achenbach System of Empirical Behavioral Assessment (ASEBA) is a standardized caregiver rating scale, assessing a broad range of behavioral, social and emotional problems, yielding standard symptom, syndrome, and composite t-scores (≥ 64 clinical range). Standardized scores for the Internalizing and Externalizing scales were used.

Services, Treatment and Intervention Data. At each visit, caregivers completed a form inquiring about current and previous intervention involvement, providing information about the type and duration of multiple types of intervention the child had received. An intensity score for each type of intervention was calculated using the following formula (weeks of intervention * hours per week)/ 168 (total hrs/week) * (number of adults/number of children). These scores were then summed. This form was adapted from the Collaborative Programs of Excellence in Autism.

Table S1. Demographic and clinical characteristics of the participants stratified on the number of IQ visits completed

|  | One Visit  (*n* = 182) | | Two visits  (*n* = 112) | | | | | Three visits  (*n* = 79) | | |
| --- | --- | --- | --- | --- | --- | --- | --- | --- | --- | --- |
|  |  |  |  |  |  |  |  |  |  |  |
|  | *n* | *Percent* | *n* | | | *Percent* | | *n* | | *Percent* |
| Male Sex^*^ | 127 | 69.8% | 68 | | | 60.7% | | 63 | | 79.8% |
| Full-scale IQ Subgroups |  |  |  | | |  | |  | |  |
| Changers | 75 | 41.2% | 40 | | | 35.7% | | 32 | | 40.5% |
| High IQ | 21 | 11.5% | 21 | | | 18.8% | | 17 | | 21.5% |
| ID | 86 | 47.3% | 51 | | | 45.5% | | 30 | | 38.0% |
| Annual Household Income |  |  |  | | |  | |  | |  |
| Under $10,000 | 4 | 2.2% | 3 | | | 2.7% | | 2 | | 2.5% |
| $10,000-$29,999 | 11 | 6.0% | 14 | | | 12.5% | | 9 | | 11.4% |
| $30,000-$49,999 | 20 | 11.0% | 15 | | | 13.4% | | 7 | | 8.9% |
| $50,000-$74,999 | 20 | 11.0% | 21 | | | 18.8% | | 20 | | 25.3% |
| $75,000-$99,999 | 32 | 17.6% | 17 | | | 15.2% | | 17 | | 21.5% |
| $100,000-$149,000 | 19 | 10.4% | 17 | | | 15.2% | | 10 | | 12.7% |
| $150,000 and above | 22 | 12.1% | 16 | | | 14.3% | | 12 | | 15.2% |
| Refused/No Response | 54 | 29.7% | 9 | | | 8.0% | | 2 | | 2.5% |
| Maternal Education |  | |  | | | | |  | | |
| None | 6 | 3.3% | 2 | | | 1.8% | | 1 | | 1.3% |
| High School or Equivalent | 62 | 34.1% | 31 | | | 27.7% | | 25 | | 31.7% |
| Bachelor’s or Associate Degree | 58 | 31.9% | 50 | | | 44.6% | | 40 | | 50.6% |
| Graduate Degree | 22 | 12.1% | 18 | | | 16.1% | | 12 | | 15.2% |
| Refused/No Response | 34 | 18.7% | 11 | | | 9.8% | | 1 | | 1.3% |
| Paternal Education |  |  |  | | |  | |  | |  |
| None | 8 | 4.4% | 5 | | | 4.5% | | 4 | | 5.1% |
| High School or Equivalent | 72 | 39.6% | 38 | | | 33.9% | | 27 | | 34.2% |
| Bachelor’s or Associate Degree | 40 | 22.0% | 38 | | | 33.9% | | 33 | | 41.8% |
| Graduate Degree | 23 | 12.6% | 20 | | | 17.9% | | 13 | | 16.5% |
| Refused/No Response | 39 | 21.4% | 11 | | | 9.8% | | 2 | | 2.5% |
|  | *n* | *Mean* (*SD*) or *Percent* | | *n* | *Mean* (*SD*) or *Percent* | | *n* | | *Mean* (*SD*) or *Percent* | |
| Mother’s Age (years) at Childbirth | 154 | 31.1 (5.5) | | 109 | 31.6 (4.8) | | 78 | | 32.6 (5.6) | |
| Father’s Age (years) at Childbirth | 138 | 33.4 (6.4) | | 101 | 34.7 (6.5) | | 71 | | 35.0 (6.3) | |
| Total Hours of Services Received at T1 | 165 | 951.1 (1202.6) | | 105 | 1026.8 (871.9) | | 76 | | 905.7 (857.2) | |
| Total Intensity of Services Received at T1 | 165 | 2168.6 (1078.3) | | 105 | 2500.8 (859.7) | | 76 | | 2296.9 (767.2) | |
| Age (years) at IQ Testing^a^ |  |  | |  |  | |  | |  | |
| T1^*^ | 182 | 3.1 (0.5) | | 112 | 3.1 (0.5) | | 79 | | 2.9 (0.5) | |
| T2 | – | – | | 75 | 5.5 (0.6) | | 79 | | 5.7 (1.0) | |
| T3^*^ | – | – | | 37 | 11.7 (1.0) | | 79 | | 11.3 (0.9) | |
| Full-scale IQ |  |  | |  |  | |  | |  | |
| T1 | 182 | 61.3 (19.1) | | 112 | 63.2 (23.3) | | 79 | | 65.3 (20.4) | |
| T2 | – | – | | 75 | 78.1 (33.0) | | 78 | | 80.0 (31.8) | |
| T3 | – | – | | 37 | 71.8 (30.5) | | 78 | | 82.2 (32.5) | |
| Verbal IQ |  |  | |  |  | |  | |  | |
| T1 | 182 | 54.1 (23.4) | | 112 | 56.3 (27.2) | | 79 | | 58.1 (25.5) | |
| T2 | – | – | | 75 | 74.2 (33.1) | | 79 | | 75.3 (33.0) | |
| T3^*^ | – | – | | 37 | 61.4 (34.7) | | 78 | | 78.8 (35.5) | |
| Nonverbal IQ |  |  | |  |  | |  | |  | |
| T1 | 182 | 68.6 (17.2) | | 112 | 70.1 (21.4) | | 79 | | 72.5 (18.1) | |
| T2 | – | – | | 75 | 81.5 (31.9) | | 79 | | 82.9 (30.8) | |
| T3 | – | – | | 35 | 78.9 (29.1) | | 78 | | 84.0 (31.0) | |
| ADOS Module Completed at T1 |  |  | |  |  | |  | |  | |
| Module 1 | 155 | 85.2% | | 95 | 84.8% | | 65 | | 82.3% | |
| Module 2 | 27 | 14.8% | | 17 | 15.2% | | 14 | | 17.7% | |
| ADOS Module Completed at T2^*^ |  |  | |  |  | |  | |  | |
| Module 1 | – | – | | 27 | 36.0% | | 24 | | 30.4% | |
| Module 2 | – | – | | 13 | 17.3% | | 29 | | 36.7% | |
| Module 3 | – | – | | 35 | 46.7% | | 26 | | 32.9% | |
| ADOS Module Completed at T3^**^ |  |  | |  |  | |  | |  | |
| Module 1 | – | – | | 13 | 35.2% | | 18 | | 23.1% | |
| Module 2 | – | – | | 8 | 21.6% | | 4 | | 5.1% | |
| Module 3 | – | – | | 16 | 43.2% | | 56 | | 71.8% | |
| ADOS-2 Calibrated Severity Score |  |  | |  |  | |  | |  | |
| T1 | 182 | 7.5 (1.7) | | 112 | 7.5 (1.7) | | 79 | | 7.4 (1.8) | |
| T2 | – | – | | 75 | 6.9 (2.2) | | 79 | | 7.2 (2.0) | |
| T3 | – | – | | 37 | 7.8 (1.7) | | 78 | | 7.5 (2.0) | |
| VABS Communication |  |  | |  |  | |  | |  | |
| T1^*^ | 162 | 72.0 (15.4) | | 105 | 71.2 (15.5) | | 75 | | 77.5 (15.8) | |
| T2 | – | – | | 60 | 79.9 (22.0) | | 72 | | 81.7 (17.7) | |
| T3 | – | – | | 35 | 67.9 (17.3) | | 75 | | 75.1 (18.4) | |
| CBCL Internalizing |  |  | |  |  | |  | |  | |
| T1 | 144 | 62.1 (10.2) | | 106 | 62.5 (9.2) | | 74 | | 61.7 (8.5) | |
| T2 | – | – | | 56 | 60.5 (10.9) | | 64 | | 58.4 (8.5) | |
| T3 | – | – | | 32 | 57.6 (10.6) | | 71 | | 61.0 (9.8) | |
| CBCL Externalizing |  |  | |  |  | |  | |  | |
| T1 | 149 | 59.2 (11.2) | | 106 | 59.7 (10.6) | | 75 | | 59.1 (10.9) | |
| T2 | – | – | | 59 | 55.8 (11.8) | | 68 | | 55.7 (8.9) | |
| T3 | – | – | | 32 | 52.7 (10.1) | | 72 | | 56.1 (9.1) | |

*Abbreviations:* SD, Standard Deviation; ADOS-2, Autism Diagnostic Observation Schedule, Second Edition; VABS, Vineland Adaptive Behavioral Scales; CBCL, Child Behavior Checklist.

^*^*p* < 0.05, ^**^*p* < 0.01, ^***^*p* < 0.001. Group differences were assesses using one-way ANOVA or Kruskal-Wallis tests (as appropriate) for continuous variables and Chi-square tests for categorical variables. Chi-square statistics are computed ignoring participants who did not respond. All statistical tests were two-tailed.

^a^All participants had IQ assessed using MSEL at T1; at T2, 28 participants were assessed using MSEL and 126 using Differential Abilities Scales-II (DAS-II) (DAS); at T3, all participants were assessed using DAS.

Table S2. Model Fit Indices for One- to Four-class Solutions in Latent Class Growth Analyses for Full-Scale IQ Scores using Quadratic Models

| Model | AIC | BIC | SBIC | BF | CMP | Entropy |
| --- | --- | --- | --- | --- | --- | --- |
| one-class | 5726 | 5753 | 5731 | – | <.0001 | – |
| two-class | 5641 | 5684 | 5649 | >1,000 | <.0001 | 0.59 |
| **three-class** | 5602 | 5660 | 5613 | >1,000 | 0.999 | 0.67 |
| four-class | 5599 | 5674 | 5613 | 0.002 | 0.002 | 0.71 |

*Abbreviations*: AIC, Akaike Information Criterion; BIC, Bayesian Information Criterion; SBIC, Sample-size adjusted Bayesian Information Criterion; BF, approximate Bayes Factor; CMP, approximate correct model probability.

The four-class solution presented a class size that was less than five percent of the sample and was therefore not considered as a candidate model. BF < 3 = weak, 3 < BF < 10 = moderate, BF > 10 = strong evidence. Any model with a CMP value > 0.10 should be considered as a candidate model.

Table S3. Comparison of Full-scale IQ Subgroups versus Solomon et al., 2018 Subgroups

|  | Full-scale IQ Subgroups | | |
| --- | --- | --- | --- |
|  |  |  |  |
|  | CHG | P-High | ID |
| Solomon et al., 2018 Subgroups |  |  |  |
| Changers (*n*=36) | 34 | 2 | 0 |
| Lesser Challenges (*n*=22) | 0 | 22 | 0 |
| High Challenges (*n*=25) | 0 | 0 | 25 |
| Stable Low (*n*=18) | 3 | 0 | 15 |

*Abbreviations:* CHG, Changers; P-High, Persistently High IQ; ID, Persistent Intellectual Disability.

Table S4. Demographic and clinical characteristics of the three IQ subgroups

|  | CHG  (*n* = 147) | | P-High  (*n* = 59) | | ID  (*n* = 167) | |
| --- | --- | --- | --- | --- | --- | --- |
|  |  |  |  |  |  |  |
|  | *n* | *Percent* | *n* | *Percent* | *n* | *Percent* |
| Male Sex | 98 | 66.7% | 40 | 67.8% | 120 | 71.9% |
| Change in ASD Diagnosis^***^ | 6^†^ | 8.3%^†^ | 4^†^ | 10.5%^†^ | 0 | 0% |
| Annual Household Income |  |  |  |  |  |  |
| Under $10,000 | 4 | 2.7% | 0 | 0 | 5 | 3.0% |
| $10,000-$29,999 | 18 | 12.2% | 0 | 0 | 16 | 9.6% |
| $30,000-$49,999 | 18 | 12.2% | 7 | 11.9% | 17 | 10.2% |
| $50,000-$74,999 | 18 | 12.2% | 15 | 25.4% | 28 | 16.8% |
| $75,000-$99,999 | 25 | 17.0% | 17 | 28.8% | 24 | 14.4% |
| $100,000-$149,000 | 23 | 15.6% | 6 | 10.2% | 17 | 10.2% |
| $150,000 and above | 19 | 12.9% | 7 | 11.9% | 24 | 14.4% |
| Refused/No Response | 22 | 15.0% | 7 | 11.9% | 36 | 21.6% |
| Maternal Education |  |  |  |  |  |  |
| None | 4 | 2.7% | 2 | 3.4% | 3 | 1.8% |
| High School or Equivalent | 42 | 28.6% | 15 | 25.4% | 61 | 36.5% |
| Bachelor’s or Associate Degree | 70 | 47.6% | 20 | 33.9% | 58 | 34.7% |
| Graduate Degree | 16 | 10.9% | 14 | 23.7% | 22 | 13.2% |
| Refused/No Response | 15 | 10.2% | 8 | 13.6% | 23 | 13.8% |
| Paternal Education |  |  |  |  |  |  |
| None | 6 | 4.1% | 2 | 3.4% | 9 | 5.4% |
| High School or Equivalent | 59 | 40.1% | 21 | 35.6% | 57 | 34.1% |
| Bachelor’s or Associate Degree | 48 | 32.7% | 20 | 33.9% | 43 | 25.7% |
| Graduate Degree | 16 | 10.9% | 8 | 13.6% | 32 | 19.2% |
| Refused/No Response | 18 | 12.2% | 8 | 13.6% | 26 | 15.6% |
|  | *n* | *Mean* (*SD*) or *Percent* | *n* | *Mean* (*SD*) or *Percent* | *n* | *Mean* (*SD*) or *Percent* |
| Mother’s Age at Childbirth (Years) | 131 | 31.9 (5.3) | 56 | 32.1 (4.9) | 154 | 31.2 (5.5) |
| Father’s Age at Childbirth (Years) | 122 | 34.6 (6.4) | 51 | 34.0 (6.1) | 137 | 33.9 (6.6) |
| Total Hours of Services Received at T1 | 137 | 1015.0 (1206.0) | 56 | 873.0 (706.6) | 153 | 951.8 (979.9) |
| Total Intensity of Services Received at T1 | 137 | 2293.0 (1003.6) | 56 | 2249.8 (808.1) | 153 | 2319.2 (978.9) |
| Age (years) at IQ Testing^a^ |  |  |  |  |  |  |
| T1^*^ | 147 | 3.1 (0.5) | 59 | 2.9 (0.4) | 167 | 3.1 (0.6) |
| T2 | 61 | 5.7 (0.8) | 34 | 5.5 (0.9) | 59 | 5.6 (0.8) |
| T3 | 43 | 11.5 (0.8) | 21 | 11.5 (0.9) | 52 | 11.4 (1.1) |
| Full-scale IQ |  |  |  |  |  |  |
| T1^***^ | 147 | 68.2 (10.6) | 59 | 96.4 (11.3) | 167 | 46.0 (10.3) |
| T2^***^ | 61 | 95.6 (13.6) | 34 | 112.1 (11.3) | 58 | 42.4 (13.9) |
| T3^***^ | 43 | 96.4 (17.6) | 20 | 111.8 (21.3) | 52 | 51.6 (20.9) |
| Verbal IQ |  |  |  |  |  |  |
| T1^***^ | 147 | 62.9 (14.9) | 59 | 93.8 (14.3) | 167 | 35.7 (13.1) |
| T2^***^ | 61 | 92.7 (14.0) | 34 | 106.4 (10.3) | 59 | 38.0 (17.5) |
| T3^***^ | 43 | 93.4 (22.1) | 21 | 108.9 (25.5) | 51 | 41.5 (19.4) |
| Nonverbal IQ |  |  |  |  |  |  |
| T1^***^ | 147 | 73.5 (10.7) | 59 | 99.1 (13.8) | 167 | 56.4 (11.0) |
| T2^***^ | 61 | 98.6 (13.2) | 34 | 113.4 (11.9) | 59 | 47.3 (14.7) |
| T3^***^ | 43 | 98.3 (16.5) | 20 | 109.5 (21.4) | 50 | 58.0 (24.0) |
| ADOS Module Completed at T1^***^ |  |  |  |  |  |  |
| Module 1 | 123 | 83.7% | 28 | 28 (47.5%) | 164 | 98.2% |
| Module 2 | 24 | 16.3% | 31 | 31 (52.5%) | 3 | 1.8% |
| ADOS Module Completed at T2^***^ |  |  |  |  |  |  |
| Module 1 | 1 | 1.6% | – | – | 50 | 84.8% |
| Module 2 | 27 | 44.3% | 7 | 7 (20.6%) | 8 | 13.6% |
| Module 3 | 33 | 54.1% | 27 | 27 (79.4%) | 1 | 1.7% |
| ADOS Module Completed at T3^***^ |  |  |  |  |  |  |
| Module 1 | – | – | – | – | 31 | 59.6% |
| Module 2 | 3 | 7.0% | – | – | 9 | 17.3% |
| Module 3 | 40 | 93.0% | 20 | 100% | 12 | 23.1% |
| ADOS-2 Calibrated Severity Score |  |  |  |  |  |  |
| T1^***^ | 147 | 7.2 (1.6) | 59 | 6.7 (1.9) | 167 | 8.1 (1.6) |
| T2^***^ | 61 | 6.9 (2.1) | 34 | 5.4 (2.1) | 59 | 8.1 (1.4) |
| T3^*^ | 43 | 7.4 (2.0) | 20 | 6.7 (2.2) | 52 | 8.1 (1.6) |
| VABS Communication |  |  |  |  |  |  |
| T1^***^ | 134 | 78.0 (12.2) | 55 | 88.9 (11.8) | 153 | 62.8 (12.3) |
| T2^***^ | 52 | 89.1 (13.8) | 33 | 94.4 (10.8) | 48 | 62.9 (16.4) |
| T3^***^ | 43 | 82.1 (13.8) | 21 | 85.4 (14.2) | 46 | 58.3 (13.3) |
| CBCL Internalizing |  |  |  |  |  |  |
| T1 | 128 | 62.0 (10.2) | 53 | 62.9 (9.9) | 143 | 62.1 (8.8) |
| T2 | 46 | 58.4 (11.5) | 30 | 60.4 (9.2) | 44 | 59.7 (7.9) |
| T3 | 42 | 61.6 (9.7) | 19 | 61.9 (12.3) | 42 | 57.4 (9.0) |
| CBCL Externalizing |  |  |  |  |  |  |
| T1 | 128 | 60.9 (11.9) | 53 | 58.4 (11.7) | 149 | 58.4 (9.5) |
| T2 | 52 | 55.3 (10.4) | 31 | 54.3 (11.1) | 45 | 57.4 (9.8) |
| T3 | 41 | 53.4 (9.4) | 21 | 55.3 (10.8) | 42 | 56.6 (8.9) |

*Abbreviations:* CHG, Changers; P-High, Persistently High IQ; ID, Persistent Intellectual Disability; SD, Standard Deviation; ADOS-2, Autism Diagnostic Observation Schedule, Second Edition; VABS, Vineland Adaptive Behavioral Scales; CBCL, Child Behavior Checklist.

^*^*p* < 0.05, ^**^*p* < 0.01, ^***^*p* < 0.001. Group differences were assesses using one-way ANOVA or Kruskal-Wallis tests (as appropriate) for continuous variables and Chi-square tests for categorical variables. Chi-square statistics are computed ignoring participants who did not respond. All statistical tests were two-tailed.

^†^ Proportion was calculated out of 72 (CHG) and 38 (P-High), respectively, the number of autistic participants with at least two timepoints.

^a^All participants had IQ assessed using MSEL at T1; at T2, 28 participants were assessed using MSEL and 126 using Differential Abilities Scales-II (DAS-II) (DAS); at T3, all participants were assessed using DAS.

Table S5. Comparison of participants who had changes of 15 points or more in full-scale, verbal, and non-verbal IQ

|  | IQ Changes of 15 points or more | | |
| --- | --- | --- | --- |
|  |  |  |  |
|  | Verbal IQ Change ≥15 | Non-verbal IQ Change ≥15 | Both |
| Changers (*n*=72) |  |  |  |
| FSIQ Change ≥15 (*n*=64) | 61 (95%) | 57 (89%) | 54 (84%) |
| FSIQ Change <15 (*n*=8) | 2 (25%) | 2 (25%) | 0 (0%) |
| P-High (*n*=38) |  |  |  |
| FSIQ Change ≥15 (*n*=22) | 17 (77%) | 16 (73%) | 12 (54%) |
| FSIQ Change <15 (*n*=16) | 4 (25%) | 2 (13%) | 0 (0%) |
| ID (*n*=81) |  |  |  |
| FSIQ Change ≥15 (*n*=18) | 15 (83%) | 13 (72%) | 11 (61%) |
| FSIQ Change <15 (*n*=63) | 4 (6%) | 2 (3%) | 0 (0%) |

*Abbreviations*: CHG, Changers; P-High, Persistently High IQ; ID, Persistent Intellectual Disability.

Table S6. Comparison of Full-scale IQ Subgroups versus Subgroups derived using NVIQ and VIQ

|  | Full-scale IQ Subgroups | | |
| --- | --- | --- | --- |
|  |  |  |  |
|  | CHG  (*n*=147) | P-High  (*n*=59) | ID  (*n*=167) |
| NVIQ Subgroups |  |  |  |
| CHG (*n*=182) | 129 (87.8%) | 25 (42.4%) | 28 (16.8%) |
| P-High (*n*=37) | 3 (2.0%) | 33 (55.9%) | 1 (0.6%) |
| ID (*n*=154) | 15 (10.2%) | 1 (1.7%) | 138 (82.6%) |
| VIQ Subgroups |  |  |  |
| CHG (*n*=157) | 13 (8.8%) | 0 (0%) | 144 (86.2%) |
| P-High (*n*=69) | 15 (10.2%) | 52 (88.1%) | 2 (1.2%) |
| ID (*n*=147) | 119 (81.0%) | 7 (11.9%) | 21 (12.6%) |

*Abbreviations*: CHG, Changers; P-High, Persistently High IQ; ID, Persistent Intellectual Disability.

Table S7. Parameter Estimates (*SE*) from the Linear Mixed-Effects Models for Clinical Characteristics

| Model term | ADOS-2 Calibrated Severity Score | | VABS Communications | | CBCL Internalizing | | CBCL Externalizing | |
| --- | --- | --- | --- | --- | --- | --- | --- | --- |
|  | Estimate (*SE*) | *p*-value | Estimate (*SE*) | *p*-value | Estimate (*SE*) | *p*-value | Estimate (*SE*) | *p*-value |
| (Intercept) | 8.17 (0.14) | <0.001 | 64.02 (1.03) | <0.001 | 62.15 (0.83) | <0.001 | 58.77 (0.94) | <0.001 |
| Age | -0.0005 (0.10) | 0.99 | -0.45 (0.74) | 0.54 | -1.78 (0.43) | <0.001 | -1.39 (0.44) | 0.002 |
| Age^2^ | -.00004 (0.01) | 0.99 | -0.04 (0.08) | 0.66 | 0.14 (0.05) | 0.003 | 0.14 (0.05) | 0.005 |
| CHG | -0.86 (0.19) | <0.001 | 15.32 (1.43) | <0.001 | -0.98 (1.13) | 0.38 | 1.76 (1.28) | 0.17 |
| CHG x Age | -0.23 (0.15) | 0.13 | 6.24 (1.07) | <0.001 | 0.68 (0.27) | 0.01 | -0.65 (0.26) | 0.01 |
| CHG x Age^2^ | 0.03 (0.02) | 0.12 | -0.59 (0.12) | <0.001 | – | – | – | – |
| P-High | -1.45 (0.24) | <0.001 | 26.51 (1.86) | <0.001 | 0.05 (1.45) | 0.98 | -0.69 (1.67) | 0.68 |
| P-High x Age | -0.60 (0.18) | 0.001 | 4.75 (1.27) | <0.001 | 0.58 (0.34) | 0.08 | -0.11 (0.32) | 0.74 |
| P-High x Age^2^ | 0.07 (0.02) | 0.001 | -0.53 (0.14) | <0.001 | – | – | – | – |
| Female Sex | -0.34 (0.17) | 0.04 | -4.18 (1.32) | 0.002 | 1.19 (1.04) | 0.26 | -0.05 (1.14) | 0.96 |

*Abbreviations:* SE, Standard Error; CHG, Changers; P-High, Persistently High IQ; ID, Persistent Intellectual Disability; ADOS-2, Autism Diagnostic Observation Schedule, Second Edition; VABS, Vineland Adaptive Behavioral Scales; CBCL; Child Behavior Checklist.

ID was used as a reference group for all mixed effects models. Age was measured in years. Each clinical characteristic was tested with a model that included fixed effects for latent class group, linear and quadratic effects of age (centered at 3 years), and sex, as well as age-by-latent class group interactions. Models also included random effects for intercept and linear and (up to) quadratic effects of age to account for within-child dependence. Higher-level interactions were tested and were not retained in the reported model if they did not contribute significantly.

Table S8. Comparison of Full-scale IQ Subgroups versus Waizbard-Bartov et al., 2022 Subgroups

|  | Full-scale IQ Subgroups | | |
| --- | --- | --- | --- |
|  |  |  |  |
|  | CHG | P-High | ID |
| Waizbard-Bartov et al., 2022 Subgroups |  |  |  |
| L-DSG; Symptom decrease (*n*=49) | 18 (25.7%) | 13 (38.2%) | 18 (23.4%) |
| L-SSG; Symptom stability (*n*=88) | 35 (50.0%) | 16 (47.1%) | 37 (48.1%) |
| L-ISG; Symptom increase (*n*=44) | 17 (24.3%) | 5 (14.7%) | 22 (28.6%) |

*Abbreviations:* CHG, Changers; P-High, Persistently High IQ; ID, Persistent Intellectual Disability. Waizbard-Bartov et al., 2022 included 182 participants, one of which is not included in the present study.

Table S9. Parameter Estimates (*SE*) from the Linear Mixed-Effects Models for Clinical Characteristics: Sensitivity Analysis

| Model term | ADOS-2 Calibrated Severity Score | | VABS Communications | | CBCL Internalizing | | CBCL Externalizing | |
| --- | --- | --- | --- | --- | --- | --- | --- | --- |
|  | Estimate (*SE*) | *p*-value | Estimate (*SE*) | *p*-value | Estimate (*SE*) | *p*-value | Estimate (*SE*) | *p*-value |
| (Intercept) | 8.07 (0.15) | <0.001 | 64.88 (1.20) | <0.001 | 61.96 (0.90) | <0.001 | 58.70 (1.01) | <0.001 |
| Age | 0.06 (0.11) | 0.58 | -1.03 (0.83) | 0.22 | -1.71 (0.44) | <0.001 | -1.39 (0.45) | 0.002 |
| Age^2^ | -0.01 (0.01) | 0.55 | 0.03 (0.09) | 0.76 | 0.14 (0.05) | 0.003 | 0.13 (0.05) | 0.005 |
| CHG | -0.62 (0.23) | 0.007 | 12.94 (1.75) | <0.001 | -0.37 (1.28) | 0.77 | 1.93 (1.50) | 0.20 |
| CHG x Age | -0.35 (0.17) | 0.04 | 7.18 (1.24) | <0.001 | 0.48 (0.30) | 0.11 | -0.60 (0.29) | 0.04 |
| CHG x Age^2^ | 0.04 (0.02) | 0.03 | -0.70 (0.14) | <0.001 | – | – | – | – |
| P-High | -1.30 (0.27) | <0.001 | 24.69 (2.12) | <0.001 | -0.13 (1.60) | 0.94 | -0.87 (1.82) | 0.63 |
| P-High x Age | -0.65 (0.19) | 0.001 | 5.65 (1.42) | <0.001 | 0.60 (0.36) | 0.09 | -0.07 (0.34) | 0.83 |
| P-High x Age^2^ | 0.08 (0.02) | 0.001 | -0.62 (0.16) | <0.001 | – | – | – | – |
| Female Sex | -0.35 (0.17) | 0.04 | -4.04 (1.45) | 0.01 | 1.16 (1.04) | 0.27 | -0.01 (1.14) | 0.99 |

*Abbreviations*: SE, Standard Error; ADOS-2, Autism Diagnostic Observation Schedule, Second Edition; VABS, Vineland Adaptive Behavioral Scales; CBCL; Child Behavior Checklist; CHG, Changers; P-High, Persistently High IQ; ID, Persistent Intellectual Disability.

ID was used as a reference group for all mixed effects models. Age was measured in years. Each clinical characteristic was tested with a model that included fixed effects for latent class group, linear and quadratic effects of age (centered at 3 years), and sex, as well as age-by-latent class group interactions. Models also included random effects for intercept and linear and (up to) quadratic effects of age to account for within-child dependence. Higher-level interactions were tested and were not retained in the reported model if they did not contribute significantly.

To account for the uncertainty in class assignments in analysis, we used 100 pseudo-class draws to randomly classify children into latent classes 100 times based on their distribution of posterior probabilities from the best fitting model, performed the analyses 100 times (i.e., for each draw) and combined results across draws using standard methods for multiple imputation for missing data.

Table S10. Summary of the Multinomial Logistic Regression Models Predicting Subgroup Membership Using Early Correlates, Intervention Intensity, and T1 to T2 Behavioral Changes: Sensitivity Analysis

|  | CHG vs ID | | | P-High vs ID | | |
| --- | --- | --- | --- | --- | --- | --- |
|  | Estimate | OR [95% CI] | *p*-value | Estimate | OR [95% CI] | *p*-value |
| Early Correlate (T1) | | | | | | |
| ADOS Severity | -0.25 | 0.78 [0.66 -0.92] | 0.004 | -0.46 | 0.63 [0.51-0.78] | <0.001 |
| VABS Communication | 0.08 | 1.09 [1.06-1.12] | <0.001 | 0.16 | 1.17 [1.12-1.22] | <0.001 |
| CBCL Internalizing | 0.003 | 1.00 [0.97-1.03] | 0.85 | 0.01 | 1.01 [0.97-1.05] | 0.64 |
| CBCL Externalizing | 0.02 | 1.02 [1.00-1.05] | 0.09 | 0.00 | 1.00 [0.97-1.03] | 0.96 |
| Total Intensity of Services Received | -0.06 | 0.94 [0.71-1.25] | 0.68 | 0.03 | 1.03 [0.72-1.46] | 0.88 |
| Behavior Change (T1 to T2) | | | | | | |
| ADOS Severity | -0.08 | 0.92 [0.76-1.13] | 0.43 | -0.31 | 0.73 [0.57-0.94] | 0.01 |
| VABS Communication | 0.08 | 1.08 [1.04-1.13] | <0.001 | 0.06 | 1.06 [1.01-1.11] | 0.01 |
| CBCL Internalizing | -0.05 | 0.96 [0.91-1.01] | 0.08 | -0.03 | 0.97 [0.92-1.03] | 0.35 |
| CBCL Externalizing | -0.08 | 0.93 [0.88-0.98] | 0.005 | -0.03 | 0.97 [0.92-1.02] | 0.20 |

*Abbreviations*: CHG, Changers; P-High, Persistently High IQ; ID, Persistent Intellectual Disability; VABS, Vineland Adaptive Behavioral Scales; CBCL, Child Behavior Checklist; OR, Odds Ratio; CI, Confidence Interval.

Early correlates are defined as scores at T1; T1 to T2 Behavior Change is defined as the difference between the score at T2 and the score at T1.

Models were adjusted for child sex and age. Estimates and OR are reported for a one-unit increase in the predictor for all variables except intensity of services received; for this variable, estimates and ORs are reported for a one-standard-deviation increase. To account for the uncertainty in class assignments in analysis, we used 100 pseudo-class draws to randomly classify children into latent classes 100 times based on their distribution of posterior probabilities from the best fitting model, performed the analyses 100 times (i.e., for each draw) and combined results across draws using standard methods for multiple imputation for missing data.
